# Trends in the Incidence of Pancreatic Adenocarcinoma in All 50 United States Examined Through an Age-Period-Cohort Analysis

**DOI:** 10.1093/jncics/pkaa033

**Published:** 2020-05-09

**Authors:** Wilson L da Costa, Abiodun O Oluyomi, Aaron P Thrift

**Affiliations:** 1 Department of Medicine, Section of Epidemiology and Population Sciences, Baylor College of Medicine, Houston, TX, USA; 2 Dan L Duncan Comprehensive Cancer Center, Baylor College of Medicine, Houston, TX, USA

## Abstract

**Background:**

Pancreatic ductal adenocarcinoma is a major contributor to cancer-related mortality in the United States. We aimed to investigate trends in incidence rates from all 50 states from 2001 to 2016, overall and by race, sex, and state and using age-period-cohort analyses.

**Methods:**

Age-adjusted incidence rates and trends in adults aged 35 years and older were calculated using data from the US Cancer Statistics registry. We used joinpoint regression to compute annual percent changes (APC) and average annual percent changes. We also analyzed incidence trends by age groups and birth cohorts through age-period-cohort modeling.

**Results:**

Age-standardized incidence rates increased by 1.23% (95% confidence interval [CI] = 0.92% to 1.54%) annually between 2001 and 2008 but were stable between 2008 and 2016 (APC = 0.11%, 95% CI = -0.13% to 0.35%). APCs and inflection points were no different for men and women. Rates increased statistically significantly among non-Hispanic whites (NHW) and non-Hispanic blacks between 2001 and 2007 and between 2001 and 2008, respectively, but, in later years, rates increased slowly among NHWs (APC = 0.36%, 95% CI = 0.12% to 0.60%), and were stable among non-Hispanic blacks (APC = -0.40%, 95% CI = -0.89% to 0.10%). The number of states with age-standardized incidence rates no less than 20.4 per 100 000 increased from 16 in 2001–2003 to 40 by 2015–2016. We found a strong birth cohort effect in both men and women and increasing rates among successive birth cohorts of NHWs.

**Conclusions:**

The incidence of pancreatic ductal adenocarcinoma has consistently increased in the United States, albeit at slower rates recently. We observed notable increases among NHWs and in some states in the central and southern part of the country.

Pancreatic cancer is currently the third-leading cause of cancer death in the United States for men and women combined (1). The American Cancer Society estimates that 56 770 new diagnoses of pancreatic cancer, along with 45 750 deaths from pancreatic cancer, are expected in the United States in 2019. According to an analysis of data from the US National Cancer Institute’s Surveillance, Epidemiology, and End Results (SEER) 9 registries, pancreatic cancer incidence increased at a rate of 0.3% per year for males and 1.0% per year for females between 2006 and 2015 (1). Assuming an average annual percent change (AAPC) in death rate of 0.5%, pancreatic cancer is expected to account for 63 000 deaths in the United States in 2030, becoming the second-leading cause of cancer death only behind lung cancer. It is also one of the two tumors (with liver cancer) expected to be associated with a steady increase in mortality in the United States (2). The vast majority of pancreatic tumors (approximately 94%) are pancreatic ductal adenocarcinomas (PDAC) (3).

Studies examining temporal trends in PDAC incidence rates in the United States have generally relied on data from either the SEER 9 (4), SEER 13 (5), or SEER 18 (6) registries (7–10). Although these SEER registries cover up to 30% of the US population (11), these data may not adequately represent overall national trends, as well as race- and ethnic-specific trends, given limited specific geographic locales and underrepresentation of minority populations in the SEER registries. Therefore, we aimed to investigate incidence rates and trends for PDAC using data from all 50 states for the period 2001– 2016, stratifying the analyses by age group, race and ethnicity, sex, and state. Furthermore, we performed age-period-cohort analyses in an effort to better discern age effects, period effects, and cohort effects on PDAC incidence rates over time.

## Methods

### Data Source and Study Population

Invasive PDAC cases from 2001 to 2016 were obtained from the US Cancer Statistics (USCS) registry (12). The USCS registry includes data collected by registries in the Centers for Disease Control’s National Program of Cancer Registries (NPCR) and National Cancer Institute’s (NCI) SEER program and provides data on 100% of cancer cases diagnosed in the United States (13). We included PDAC cases defined by the SEER*Stat software version 8.3.6 (14) site recode International Classification of Diseases for Oncology, 3rd edition (ICD-O-3)–World Health Organization 208: Pancreas (ICD-O-3 site C25). Pancreatic neuroendocrine tumors (ICD-O-3 histology codes 8150–8156, 8240, and 8246) were not included. We used incidence data for patients who were 35 years or older at cancer diagnosis because of the small number of cases per year among subjects younger than 35 years and the SEER–NPCR practice of compressing cancer counts less than 16 cases per year. Results were stratified for both sexes, and for non-Hispanic whites (NHW), non-Hispanic blacks (NHB), and Hispanics. Other races and ethnicities were not analyzed because of small numbers of PDAC cases.

### Statistical Analysis

Annual incidence rates for PDAC were calculated using formulae implemented in SEER*Stat, using the number of cases as the numerator and the population size (based on US Census Bureau Data) as the denominator (12). Associated 95% confidence intervals (CI) were calculated using the Tiwari method (15). We present age-group-specific and age-standardized (2000 US standard population) incidence rates.

To estimate annual percent change (APC) in PDAC incidence rates, we fit a least-squares regression line to the natural logarithm of the incidence rate, using the year of diagnosis as a regressor variable. A maximum of 2 joinpoints with a minimum of 4 observations required between those joinpoints were allowed (16). We used Monte Carlo permutation tests to examine trends for each joinpoint combination, and the trend line that best fitted the data was selected (17). APC values for each linear segment were estimated, as were AAPC values for the study period between 2001 and 2016, using Joinpoint Trend Analysis software (18). The AAPC was calculated using a weighted average of the slope coefficients of the underlying joinpoint regression line with the weights equal to the length of each segment over the interval (19). A parallelism test was used to examine whether the slopes of the change in trend between groups were similar in direction. A statistically significant *P* value on this test indicates that the 2 trends in terms of AAPCs compared were statistically significantly different from each other (20). All tests were two-sided with a statistical significance level of α = 0.05.

We calculated state-specific, age-adjusted PDAC incidence rates for 4 separate time periods—2001–2002, 2005–2006, 2010–2011, and 2014–2015—and assessed geographic trends in the incidence rates over the entire study period in all states through choropleth maps created in ArcGIS Pro 2.0 (Esri, Redlands). We used the same class breaks shared among all 4 maps (time periods), making the overall increase in rates through time more obvious (21,22). We first established the minimum (14.5) and maximum (26.1) incidence rates across the 4 time periods. Thereafter, we divided the range (26.1–14.5 = 11.6) by the number of classes that we used for our maps (11.6/4 = 2.9). We then allowed each class to maintain intraclass interval of 2.9, starting with 14.5–17.4 as the first class, and 20.4 was the median of incidence rate over the study period.

Finally, we used age-period-cohort models to search for patterns in secular incidence trends accounting for age at diagnosis (age), year of diagnosis (period), and year of birth (cohort). These models were fit using the NCI’s Age-Period-Cohort web tool, which provided estimates of net drifts (APC in expected age-adjusted rates over time), local drifts (APC in expected age-specific rates over time), and cohort rate ratios (ratio of age-specific rates in each birth cohort relative to the reference cohort) and enabled testing of equality of observed trends (23). We used 11 five-year age groups (35–39 years through 85 years and older), and 4 four-year calendar periods (2001–2004 through 2013–2016). Default reference groups were used for comparisons (ie, calendar period, 2005–2008, and birth cohort, 1946).

## Results

### Overall Trends

Between 2001 and 2016, 576 301 persons were diagnosed with PDAC in the United States according to the USCS registry. New cases increased from 28 554 in 2001 to 43 175 in 2016—an increase of 51.2% (Table 1). The age-adjusted rate for the entire study period was 21.4 per 100 000 (95% CI = 21.3 to 21.5), increasing from 20.0 per 100 000 (95% CI = 19.8 to 20.2) in 2001 to 21.7 per 100 000 (95% CI = 21.5 to 22.0) in 2016. The AAPC in age-adjusted incidence rates of PDAC from 2001 to 2016 was 0.63% (95% CI = 0.46% to 0.80%). Joinpoint regression identified one statistically significant inflection point (2008). Age-standardized incidence rates for PDAC increased by 1.23% (95% CI = 0.92% to 1.54%) annually between 2001 and 2008 but remained stable between 2008 and 2016 (APC = 0.11%, 95% CI = -0.13% to 0.35%) (Table 2).

**Table 1.** Annual frequencies and age-adjusted incidence rates of pancreatic adenocarcinoma in the United States between 2001 and 2016^a^

Year	Incident pancreatic adenocarcinoma	Age-adjusted rate per 100 000 (95% CI)
2001	28 554	20.0 (19.8 to 20.2)
2002	28 975	20.0 (19.7 to 20.2)
2003	30 707	20.6 (20.3 to 20.8)
2004	31 528	20.7 (20.5 to 21.0)
2005	32 615	21.0 (20.8 to 21.3)
2006	33 572	21.3 (21.1 to 21.5)
2007	34 511	21.4 (21.2 to 21.7)
2008	36 022	21.9 (21.6 to 22.1)
2009	36 375	21.6 (21.4 to 21.8)
2010	37 259	21.6 (21.4 to 21.9)
2011	38 399	21.8 (21.6 to 22.1)
2012	39 680	22.0 (21.7 to 22.2)
2013	40 283	21.8 (21.5 to 22.0)
2014	41 703	22.0 (21.8 to 22.2)
2015	42 943	22.1 (21.9 to 22.4)
2016	43 175	21.7 (21.5 to 22.0)

^a^CI = confidence interval.

**Table 2.** Annual percent change (APC) and average annual percent change (AAPC) in pancreatic cancer incidence rates over time among the US population older than 35 years, overall and by age, sex, and race

Population	Joinpoint segment			Joinpoint segment			
	Year start	Year end	APC (95% CI), %	Year start	Year end	AAPC (95% CI), %	*P*
Overall US population	2001	2008	1.23 (0.92 to 1.54)	2001	2016	0.63 (0.46 to 0.80)	
	2008	2016	0.11 (-0.13 to 0.35)				
Age-group at diagnosis, y							
35–39	2001	2013	–0.58 (–1.62 to 0.47)	2001	2016	1.58 (–0.00 to 3.18)	.018
	2013	2016	10.68 (2.46 to 19.55)				
40–44	2001	2007	1.70 (–0.01 to 3.44)	2001	2016	0.66 (–0.66 to 1.93)	<.001
	2007	2013	–1.78 (–3.89 to 0.37)				
	2013	2016	3.59 (–1.39 to 8.81)				
45–49	2001	2006	1.68 (0.81 to 2.56)	2001	2016	0.44 (0.13 to 0.76)	.001
	2006	2016	–0.17 (–0.48 to 0.14)				
50–54	2001	2016	0.72 (0.45 to 0.99)	2001	2016	0.72 (0.45 to 0.99)	.757
55–59	2001	2016	0.88 (0.63 to 1.13)	2001	2016	0.88 (0.63 to 1.13)	.329
60–64	2001	2016	0.82 (0.56 to 1.07)	2001	2016	0.82 (0.56 to 1.07)	.638
64–69	2001	2016	0.76 (0.59 to 0.93)	2001	2016	0.76 (0.59 to 0.93)	^a^
70–74	2001	2008	1.13 (0.66 to 1.60)	2001	2016	0.64 (0.39 to 0.89)	.011
	2008	2016	0.21 (–0.12 to 0.55)				
75–79	2001	2009	1.30 (0.91 to 1.69)	2001	2016	0.72 (0.46 to 0.98)	.155
	2009	2016	0.06 (–0.39 to 0.50)				
80–84	2001	2006	2.35 (0.77 to 3.96)	2001	2016	0.83 (0.28 to 1.38)	.126
	2006	2016	0.08 (–0.42 to 0.58)				
≥85	2001	2008	1.56 (0.50 to 2.63)	2001	2016	–0.01 (–0.57 to 0.55)	.002
	2008	2016	–1.37 (–2.10 to –0.63)				
Sex							
Men	2001	2008	1.14 (0.71 to 1.58)	2001	2016	0.61 (0.42 to 0.81)	^a^
	2008	2016	0.26 (0.04 to 0.48)				
Women	2001	2008	1.35 (1.00 to 1.70)	2001	2016	0.63 (0.44 to 0.82)	.250
	2008	2016	0.01 (–0.26 to 0.27)				
Race/Ethnics							
Non–Hispanic White	2001	2007	1.47 (1.00 to 1.95)	2001	2016	0.80 (0.59 to 1.01)	^a^
	2007	2016	0.36 (0.12 to 0.60)				
Non–Hispanic Black	2001	2008	1.19 (0.46 to 1.93)	2001	2016	0.34 (–0.04 to 0.73)	<.001
	2008	2016	–0.40 (–0.89 to 0.10)				
Hispanic	2001	2016	0.07 (–0.22 to 0.36)	2001	2016	0.07 (–0.22 to 0.36)	.003

^a^ Reference group for comparisons of incidence trends. Age groups were compared with age group 64–69 years. Non–Hispanic blacks and Hispanics were compared with non–Hispanic whites. *P* value < .05 on the parallelism test, indicating that the trends (slopes) between this reference groups were statistically significantly different. CI = confidence interval.

### Age

The highest age-specific incidence rates for PDAC were observed among persons aged 75–79 years (69.4 per 100 000), 80–84 years (80.0 per 100 000), and 85 years and older (81.9 per 100 000). Age-specific incidence rates increased with increasing age for all age-groups of persons aged 50–84 years; we found no statistically significant trends among persons younger than 50 years. The trends in incidence of PDAC over time varied across age groups. Among persons aged 50–69 years, PDAC incidence increased linearly between 2001 and 2016, with AAPC values between 0.72% and 0.88% (Table 2). For persons aged 70–84 years, PDAC incidence increased at a rate of 1.13%–2.35% annually between 2001 and the mid-2000s, with subsequent stabilization in more recent years. The trends among persons aged 35–49 years were mixed and imprecise owing to the smaller number of cases.

### Sex

There were increases in age-standardized incidence rates for PDAC in both men and women over the study period. Among men, incidence rates increased from 23.0 per 100 000 in 2001 to 24.8 per 100 000 in 2016. Among women, incidence rates increased from 17.6 per 100 000 in 2001 to 19.2 per 100 000 in 2016. Joinpoint regression analysis showed similar AAPCs in men and women (0.61% and 0.63%, respectively; *P* value parallelism test = 0.25). We observed annual increases of 1.14% (95% CI = 0.71% to 1.58%) among men and 1.35% (95% CI = 1.00% to 1.70%) among women between 2001 and 2008. After that, a statistically significant but smaller increase was found among men (APC = 0.26%, 95% CI = 0.04% to 0.48%), but rates remained stable among women (APC = 0.01%, 95% CI = -0.26% to 0.27%) (Table 2).

### Race and Ethnicity

Parallelism tests indicated that incidence trends were statistically significantly different between NHWs and NHBs (*P*  < .001) and NHWs and Hispanics (*P*  = .003). Among NHWs, PDAC incidence increased from 19.6 per 100 000 in 2001 to 21.7 per 100 000 in 2016. The rate of increase was 1.47% per year (95% CI = 1.00% to 1.95%) between 2001 and 2007; the rate of increase slowed but remained statistically significant in subsequent years (APC = 0.36%, 95% CI = 0.12% to 0.60%). Among NHBs, PDAC incidence rates were still the highest, but they were mostly stable, from 27.3 per 100 000 in 2001 to 26.5 per 100 000 in 2016. They increased by 1.19% per year (95% CI = 0.46% to 1.93%) between 2001 and 2008 but remained stable between 2008 and 2016 (APC = -0.40%, 95% CI = -0.89% to 0.10%). We observed no change in the incidence of PDAC among Hispanics between 2001 and 2016 (AAPC = 0.07%, 95% CI = -0.22% to 0.36%).

### Geography

**Figure 1.**
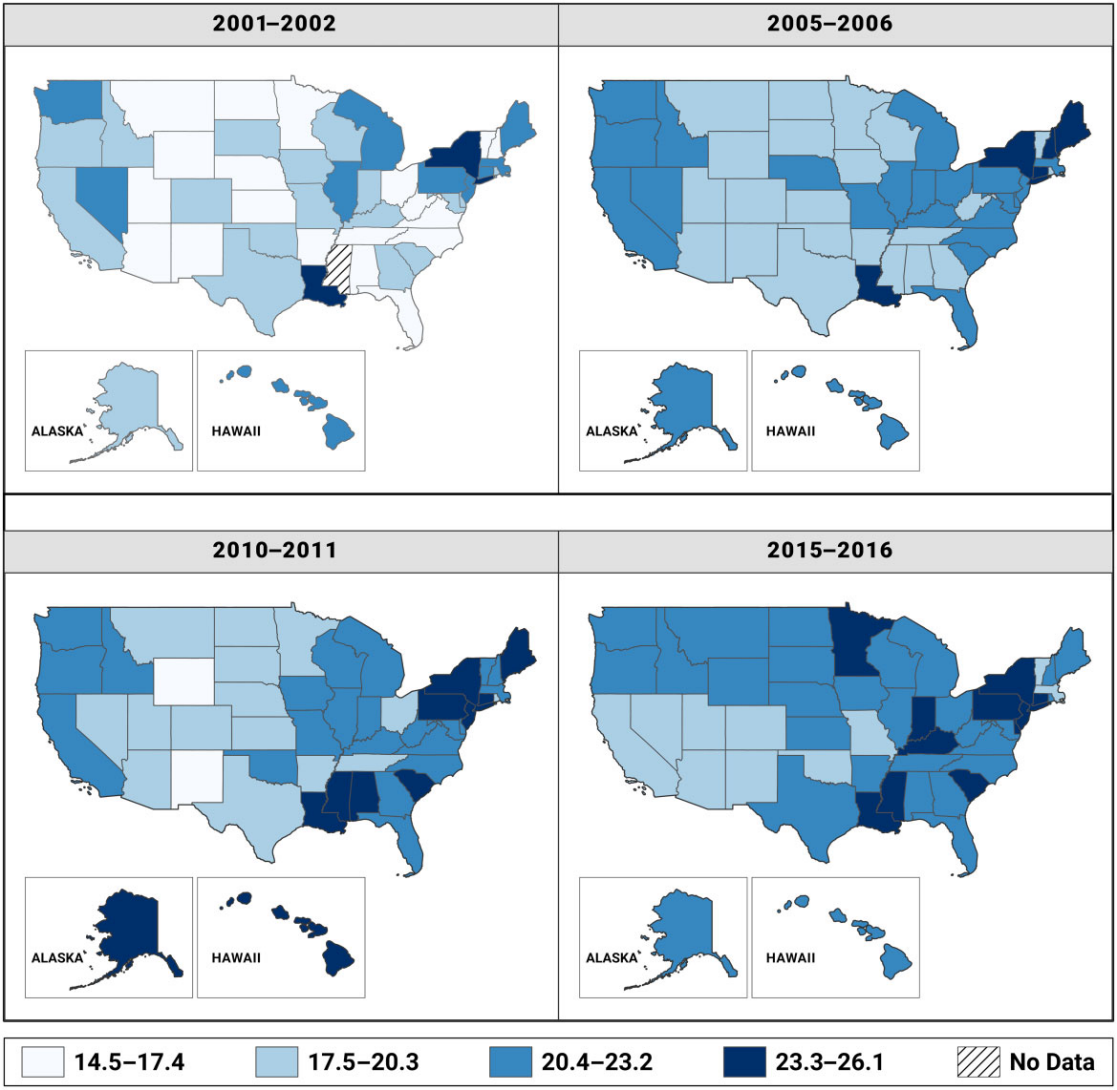
State-level heat maps showing age-adjusted PDAC incidence rates in the United States in 2001–2002, 2005–2006, 2010–2011, and 2014–2015.

Interesting findings were noted when age-adjusted incidence rates and trends were analyzed by state. The highest age-standardized incidence rates for PDAC were found in Louisiana (24.3 per 100 000), New York (24.1 per 100 000), and Connecticut (24.0 per 100 000). In 2001–2002, 16 of the 50 states had age-standardized incidence rates no less than 20.4 per 100 000; this number increased to 28 states by 2005–2006, 33 states by 2010–2011, and 40 states by 2015–2016 (Figure 1). In contrast, the number of states with age-standardized incidence rates less than 17.5 per 100 000 decreased from 7 states in 2001–2002 to 0 states by 2015–2016. Incidence rates by state are provided in Supplementary Tables 1–4 (available online). Trends, however, were not consistent across states. In California, incidence rates were stable between 2001 and 2008, but a statistically significant decreasing trend was observed between 2008 and 2016 (APC = -1.20%, 95% CI = -1.69% to -0.71%). In New York and Texas, an overall increasing trend was found, although the former had a stable trend after 2009. Finally, several states had increasing trends, including Mississippi (AAPC = 2.99%), Minnesota (AAPC = 2.66%), Tennessee (AAPC = 2.21%), Nebraska (AAPC = 1.58%), and Alabama (AAPC = 1.52%). State-specific trends among only NHWs were similar to the overall results; however, there were too few cases by state for NHBs and Hispanics for meaningful comparisons (Supplementary Tables 5–8, available online).

### Age-Period-Cohort Models

**Figure 2.**
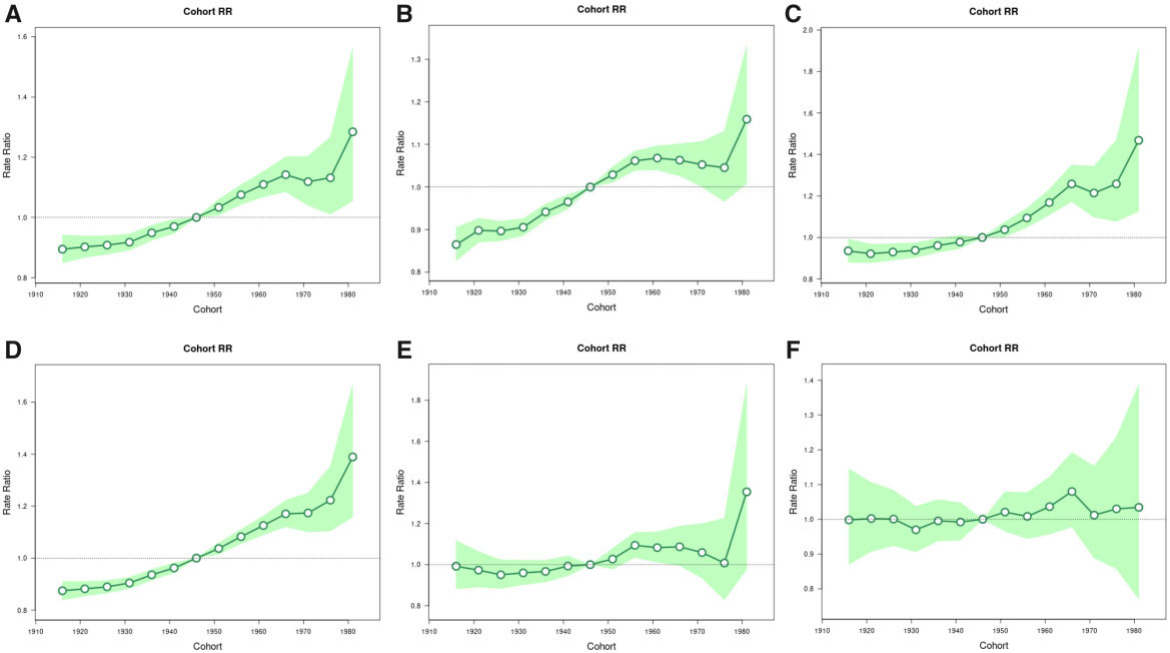
Birth cohort rate ratios of PDAC incidence rates in the United States. **A)** All PDAC; **(B)** Males; **(C)** Females; **(D)** Non–Hispanic whites; **(E)** Non–Hispanic blacks; **(F)** Hispanics. PDAC = pancreatic ductal adenocarcinomas; RR = rate ratios.

Age-period-cohort analysis identified both period and cohort effects, with a particularly striking cohort effect (Figure 2). Age-specific trends by birth cohort are presented as incidence rate ratios (IRR) using the 1946 cohort as the reference group. Among men and women, rates increased linearly across birth cohorts with the highest rates among the most recent birth cohorts. Compared with men and women born circa 1946, PDAC incidence rates were 1.16 (95% CI = 1.01 to 1.33) times higher among men and 1.47 (95% CI = 1.13 to 1.96) times higher among women born circa 1981. A progressive increase in IRRs was found among every NHW birth cohort compared with those born circa 1946 from an IRR of 1.04 (95% CI = 1.02 to 1.06) among those born circa 1951 to an IRR of 1.39 (95% CI = 1.16 to 1.67) among those born circa 1981. Conversely, there was little evidence for a cohort effect among NHBs and Hispanics (Figure 2).

## Discussion

In our population-based study, we observed that PDAC incidence rates in the United States increased by 1.23% per year between 2001 and 2008 but remained stable between 2008 and 2016. Similar trends in PDAC incidence rates were observed for men and women (increasing until 2008 and remaining relatively stable thereafter); however, trends varied by age group, race and ethnicity, and state. In age-period-cohort analyses, we observed a strong cohort effect among NHWs, with increasing incidence rates among recent and current birth cohorts. Conversely, we found little evidence for a cohort effect on PDAC trends among NHBs and Hispanics.

Results from our national level analysis support those from previous studies showing increasing incidence of PDAC in the United States. Both our analysis and previous trends from SEER 13 include inflection points that suggest a more statistically significant increase until the mid-2000s (24). It also confirms the impact of age on PDAC in which incidence rates have increased among almost all age groups. The differences were related to how they increased among each age group, with patients 70 years and older showing a marked increase in the early calendar years, and those between 50 and 69 years having a slower but constant increase over the study period. A recent study that focused on young patients used age-period-cohort analysis to investigate the incidence of several tumors in the United States between 1995 and 2014, and it identified an APC for PDAC of 4.34% for persons aged 25–29 years (25).

Our study also identified several disparities and changes in trends of pancreatic cancer incidence. The first concerns its distribution by sex. While prior studies have reported a male to female ratio in incidence of 2:1, we observed much more similar rates among males and females for the period 2001–2016 (26). Our data is, however, consistent with the most recent estimations from the American Cancer Society showing only a small male predominance in incidence (29 940 new cases among men and 26 830 among women) (1,9). We found that trends and rates of change in PDAC incidence in the United States were similar for males and females. The strong birth cohort effect also observed in males and females suggests that increasing incidence of PDAC in both sexes is because of changes in the prevalence of exposure to causal factors, which differ across successive generations but have equally impacted males and females.

Incidence trends of PDAC analyses also identified marked racial disparities. Highest incidence rates, along with higher mortality rates and metastatic disease at diagnosis, have been described among NHBs (8,27). In a study focused on racial disparities associated with PDAC, between 2001 and 2015, PDAC incidence was 1.28 times higher among NHBs compared with NHWs, and mortality was 1.27 times higher (27). Our study confirmed the higher incidence rates among NHBs but suggests PDAC rates were stable and might even be decreasing among NHBs in recent years. This confirms findings from previous studies concerning NHBs, which failed to identify annual changes in incidence among this population between 2003 and 2012 and showed a 0.5% annual decrease in mortality (24). Analyses of a smaller population over a longer period, between 1973 and 2014, also showed a decreasing trend in the incidence of the disease among NHBs, whereas among NHWs, incidence increased 0.9% annually between 1994 and 2014 (28).

One of our most impactful findings concerns marked disparities in incidence trends among states. In the most populous states, incidence trends are either stable or declining (California and New York) or showing slight increases (Florida and Texas). On the contrary, states in the central and southeastern parts of the country have shown marked increases, which were not related to age, sex, or race and ethnic groups. Previous data stratified by state have focused either on the absolute number of cases or on racial disparities in incidence and mortality (1,27). The higher incidence trends we identified in several states showed how this disease is growing in some areas independently of other known disparities.

Divergent trends in prevalence of the main risk factors for PDAC in the US population may provide at least a partial explanation for our findings. Given that smoking confers an approximate twofold increased risk for PDAC, it is anticipated that the decline in smoking may also have removed one of the disease’s drivers. Conversely, dramatic increases followed by stabilization of obesity prevalence rates may help explain the increasing trends through to 2008 and subsequent largely stable incidence rates since 2008. Likewise, increasing prevalence of diabetes and subsequent declining rates may have contributed to increasing PDAC incidence and recent stabilization. Smoking, obesity, and diabetes rates may help explain the more similar rates in recent decades among males and females and also the similar trends (29). Finally, states with higher AAPCs in our analyses are generally those states in the United States with a history of either very high smoking rates (most southeastern states) or high obesity and sugar-sweetened beverages consumption rates or which have higher prevalence of diabetes (30–34).

We believe one major strength of our study is the use of the SEER–NPCR database, which includes the entire US population instead of the other SEER databases, which are more commonly used in studies of PDAC incidence. There is also a very low risk of recall or information bias, because data was collected prospectively and independently of our study. Furthermore, one of our main findings, the disparities among states, would not have been possible if other data sources were used. Finally, our findings on PDAC are relevant for risk factor control and hypotheses development. Despite a modest overall annual increase of 0.6%, we found relevant disparities among different ages, races, and states. Known risk factors might explain some of these findings, but more research is needed, especially regarding the impact of diet, obesity, and other environmental exposures on the cohort effects we observed.

Our study’s main limitation concerns the fact that some of our findings were observed among smaller subgroups of patients, such as younger populations, or those from less populated states. Still, we believe that these disparities confirm that even though PDAC incidence increase has slowed in recent years, it still affects several populations across states. In addition, the association between these trends and those related to some of this disease’s main risk factors, such as smoking, and perhaps obesity, reinforces the validity of our findings and stresses the demand on additional knowledge concerning the main causes of some of these trends.

In summary, our study identified that overall PDAC incidence rates have increased in the United States in the period between 2001 and 2016, most statistically significantly among patients 70 years or older and similarly among males and females. Although the overall rate of increase may have slowed, the persistent and strong cohort effect observed among NHWs and the increasing rates among some individual states suggests that PDAC rates will continue to rise in the United States in the near future. Increasing focus needs to be directed toward the detection and treatment of underlying risk factors for PDAC.

### Funding

Wilson L. da Costa Jr was funded by a Research Training Award for Cancer Prevention Post-Graduate Training Program in Integrative Epidemiology from the Cancer Prevention and Research Institute of Texas (CPRIT; RP160097; PI: M. Spitz).

### Notes

**Role of the funder:** The funder had no role in the design of the study; the collection, analysis, and interpretation of the data; the writing of the manuscript; and the decision to submit the manuscript for publication.

**Conflicts of interest:** No relevant conflicts of interest exist. 

**Author contributions:** WLDCJ was involved in the conceptualization, data curation, data analysis, writing. AO was contributed to the methodology, data analysis, manuscript review. APT was involved in the conceptualization, methodology, data analysis, writing, manuscript review.

## Supplementary Material

pkaa033_Supplementary_Data
