# Supplementary material for: Trends in the Incidence of Pancreatic Adenocarcinoma in All 50 United States Examined Through an Age-Period-Cohort Analysis
Source: JNCI Cancer Spectr. 2020 May 9;4(4):pkaa033. doi: 10.1093/jncics/pkaa033 (PMC7365041; doi:10.1093/jncics/pkaa033)
Supplement: pkaa033_Supplementary_Data [file pkaa033_supplementary_data.pdf]

Supplementary Table 1. Age-standardized incidence rates of pancreatic cancer by state (2001-2002)

State	Count	Pop	Rate/100,000	Lower CI	Upper CI
Louisiana	1,018	4,423,452	23.7	22.2	25.2
New York	4,674	19,890,788	23.1	22.5	23.8
Washington	1,295	6,139,087	22.5	21.3	23.8
Maine	338	1,461,892	22.3	20.0	24.8
Michigan	2,239	10,274,658	22	21.1	22.9
New Jersey	2,008	9,120,732	21.9	21.0	22.9
Connecticut	841	3,763,819	21.8	20.3	23.3
Hawaii	290	1,294,759	21.7	19.3	24.3
Illinois	2,693	12,500,868	21.7	20.9	22.6
Nevada	434	2,162,084	21.7	19.7	23.9
Massachusetts	1,499	6,811,832	21.5	20.4	22.6
Pennsylvania	3,175	13,559,593	21.1	20.4	21.8
South Carolina	851	4,191,837	20.7	19.4	22.2
Wisconsin	1,187	5,661,681	20.6	19.4	21.8
California	6,278	33,154,880	20.4	19.9	20.9
Rhode Island	249	1,130,154	20.4	17.9	23.1
Maryland	1,058	5,655,877	20.3	19.1	21.6
Georgia	1,414	8,087,697	20.2	19.1	21.2
Indiana	1,256	6,233,201	20.1	19.0	21.3
Iowa	695	3,094,272	20.1	18.6	21.7
Oregon	747	3,648,698	20.1	18.7	21.6
Idaho	248	1,277,940	19.8	17.4	22.4
Alaska	80	597,931	19.7	15.3	24.9
Kentucky	827	4,234,003	19.7	18.3	21.1
Colorado	717	4,379,904	19.4	18.0	20.9
Delaware	163	838,679	19.3	16.4	22.5
Oklahoma	715	3,545,353	19.3	18.0	20.8
Missouri	1,182	5,894,767	19.2	18.1	20.3
South Dakota	162	778,557	19.0	16.2	22.2
Texas	3,423	19,939,834	19.0	18.4	19.7
Arkansas	567	2,802,528	18.9	17.4	20.5
Florida	4,097	18,541,901	18.9	18.4	19.5
North Carolina	1,556	8,432,376	18.9	18.0	19.9
New Hampshire	239	1,369,826	18.9	16.6	21.4
Alabama	892	4,639,484	18.8	17.6	20.1
Wyoming	95	519,084	18.8	15.2	23.0
Ohio	2,293	11,936,943	18.7	17.9	19.4
Vermont	123	674,454	18.4	15.3	22.0
Utah	296	1,808,306	17.9	15.9	20.1

Arizona	981	5,247,480	17.8	16.7	19
Nebraska	332	1,750,645	17.6	15.8	19.6
West Virginia	389	2,024,619	17.6	15.9	19.4
Kansas	503	2,737,853	17.5	16.0	19.1
New Mexico	310	1,844,585	17.0	15.2	19.0
North Dakota	127	665,162	16.9	14.0	20.1
Tennessee	923	5,996,756	15.5	14.5	16.6
Minnesota	748	5,108,965	15.2	14.1	16.3
Montana	149	983,340	14.6	12.4	17.2
Virginia	1,009	7,489,079	14.5	13.6	15.4
Mississippi	^	0	^	^	^

Supplementary Table 2. Age-standardized incidence rates of pancreatic cancer by state (2005-2006)

State	Count	Pop	Rate/100,000	Lower CI	Upper CI
New Hampshire	349	1,461,566	24.7	22.2	27.5
Connecticut	988	3,911,582	24.5	23.0	26.1
Louisiana	1,056	4,428,283	24.0	22.6	25.5
Maine	388	1,537,952	23.8	21.5	26.3
New York	4,963	20,395,482	23.6	22.9	24.2
Illinois	3,007	12,916,744	23.2	22.4	24.0
Massachusetts	1,672	6,987,919	23.2	22.1	24.4
Michigan	2,515	10,631,792	23.2	22.3	24.1
New Jersey	2,168	9,435,742	22.7	21.8	23.7
Hawaii	334	1,393,963	22.5	20.2	25.1
Pennsylvania	3,476	13,937,120	22.4	21.7	23.2
Washington	1,419	6,621,528	22.3	21.1	23.5
Delaware	209	914,731	22.2	19.3	25.5
Virginia	1,681	8,015,737	22.1	21.1	23.2
Alaska	94	645,458	22.0	17.5	27.3
Missouri	1,424	6,147,602	21.9	20.8	23.1
Kentucky	985	4,457,975	21.8	20.5	23.3
South Carolina	1,002	4,540,210	21.6	20.3	23.0
California	7,174	35,346,994	21.5	21.0	22.1
Maryland	1,207	5,965,750	21.5	20.3	22.8
Ohio	2,749	12,291,386	21.3	20.5	22.1
Idaho	300	1,413,998	21.0	18.7	23.5
North Carolina	1,911	9,225,228	20.9	19.9	21.8
Nebraska	406	1,811,648	20.8	18.8	22.9
Florida	4,864	20,344,029	20.7	20.1	21.2
Indiana	1,368	6,511,882	20.6	19.5	21.8
Nevada	494	2,535,715	20.6	18.8	22.6
Oregon	829	3,907,357	20.4	19.0	21.8
Montana	226	1,038,185	20.3	17.7	23.1
Vermont	142	703,876	20.2	17.0	23.8
Alabama	1,007	4,858,613	19.9	18.7	21.2
Mississippi	593	2,910,710	19.9	18.3	21.6
Georgia	1,567	8,902,801	19.8	18.8	20.8
Iowa	709	3,178,583	19.8	18.3	21.3
North Dakota	151	678,969	19.7	16.6	23.1
Oklahoma	762	3,679,009	19.5	18.2	21.0
Wyoming	107	546,494	19.5	15.9	23.6
Wisconsin	1,190	5,938,726	19.4	18.3	20.6
New Mexico	394	1,991,898	19.3	17.4	21.3

Arkansas	610	2,940,335	19.2	17.7	20.8
Texas	3,835	21,840,120	19.2	18.6	19.8
Rhode Island	234	1,158,791	18.9	16.5	21.5
Utah	351	2,000,550	18.9	16.9	21.0
West Virginia	441	2,083,875	18.9	17.1	20.7
Colorado	774	4,733,278	18.4	17.1	19.8
Kansas	548	2,823,534	18.2	16.7	19.8
Tennessee	1,173	6,396,848	18.1	17.1	19.2
Minnesota	946	5,367,362	17.9	16.7	19.0
South Dakota	162	810,908	17.6	15.0	20.6
Arizona	1,105	5,941,517	17.5	16.5	18.6

Supplementary Table 3. Age-standardized incidence rates of pancreatic cancer by state (2010-2011)

State	Count	Pop	Rate/100,000	Lower CI	Upper CI
Louisiana	1,219	4,657,693	25.0	23.6	26.4
New York	5,564	20,931,659	24.7	24.1	25.4
Connecticut	1,059	4,032,113	24.4	22.9	25.9
Mississippi	791	3,054,381	24.4	22.7	26.2
Maine	434	1,589,296	24.2	21.9	26.6
Pennsylvania	4,009	14,301,885	24.2	23.5	25.0
Alaska	130	695,564	24.1	19.8	29.0
New Jersey	2,457	9,747,164	24.0	23.0	25.0
Hawaii	402	1,491,568	23.9	21.5	26.4
South Carolina	1,278	5,000,920	23.9	22.6	25.3
Alabama	1,331	5,143,690	23.7	22.4	25.0
Kentucky	1,150	4,699,261	23.1	21.8	24.5
Missouri	1,627	6,420,233	22.9	21.8	24.0
Wisconsin	1,540	6,176,853	22.9	21.7	24.1
Illinois	3,175	13,427,997	22.8	22.0	23.6
Maryland	1,432	6,250,865	22.8	21.6	24.0
Michigan	2,690	10,819,689	22.8	21.9	23.7
Washington	1,638	7,188,931	22.5	21.4	23.7
Delaware	245	987,669	22.1	19.4	25.1
Indiana	1,614	6,829,337	22.1	21.0	23.2
Iowa	826	3,269,628	21.7	20.3	23.3
Vermont	166	724,943	21.6	18.4	25.3
California	8,047	37,814,331	21.4	21.0	21.9
Georgia	2,000	9,890,163	21.4	20.5	22.4
Idaho	359	1,566,909	21.4	19.2	23.8
Oregon	984	4,198,720	21.4	20.1	22.8
North Carolina	2,260	10,244,298	21.0	20.2	21.9
Massachusetts	1,634	7,250,826	20.9	19.9	22.0
Florida	5,476	21,771,504	20.8	20.2	21.3
New Hampshire	325	1,527,690	20.6	18.4	23.0
Oklahoma	878	3,887,739	20.6	19.3	22.0
Virginia	1,789	8,584,394	20.6	19.7	21.6
West Virginia	508	2,154,676	20.4	18.6	22.2
Texas	4,745	24,538,208	20.3	19.7	20.9
Arizona	1,464	6,624,485	19.9	18.9	21.0
Montana	250	1,100,977	19.9	17.5	22.6
Nebraska	416	1,887,751	19.9	18.0	22.0
South Dakota	199	853,536	19.9	17.2	23.0
Ohio	2,738	12,615,947	19.7	18.9	20.4

Tennessee	1,456	6,886,260	19.6	18.6	20.6
Utah	426	2,301,777	19.4	17.5	21.3
Colorado	963	5,241,301	19.1	17.9	20.4
Rhode Island	248	1,163,875	19.0	16.6	21.6
Minnesota	1,125	5,638,597	18.9	17.8	20.1
North Dakota	161	705,503	18.9	16.1	22.2
Arkansas	652	3,106,208	18.7	17.3	20.2
Kansas	597	2,931,589	18.7	17.2	20.3
Nevada	530	2,825,236	18.6	17.0	20.3
New Mexico	400	2,153,030	17.0	15.3	18.7
Wyoming	98	591,173	15.2	12.3	18.6

Supplementary Table 4. Age-standardized incidence rates of pancreatic cancer by state (2015-2016)

State	Count	Pop	Rate/100,000	Lower CI	Upper CI
Mississippi	929	3,139,618	26.1	24.5	27.9
Louisiana	1,396	4,865,507	25.8	24.4	27.2
Connecticut	1,193	4,082,857	25.0	23.5	26.4
Pennsylvania	4,348	14,483,743	24.6	23.9	25.4
New York	6,043	21,629,168	24.5	23.8	25.1
Delaware	313	1,061,131	24.4	21.7	27.4
South Carolina	1,497	5,420,216	23.7	22.5	25.0
Indiana	1,896	7,055,748	23.6	22.6	24.8
Kentucky	1,291	4,845,676	23.4	22.1	24.7
New Jersey	2,630	10,054,703	23.4	22.5	24.4
Minnesota	1,551	5,922,242	23.3	22.1	24.5
Maine	463	1,616,010	23.2	21.1	25.5
Wisconsin	1,718	6,372,410	23.2	22.1	24.3
Idaho	448	1,710,019	23.1	21.0	25.4
Michigan	2,998	11,013,989	23.1	22.3	24.0
South Dakota	251	899,034	23.1	20.3	26.3
Alabama	1,398	5,311,916	22.8	21.6	24.1
Nebraska	516	1,965,549	22.8	20.9	25.0
Illinois	3,482	13,800,154	22.7	22.0	23.5
Iowa	916	3,358,591	22.6	21.1	24.2
Alaska	150	717,210	22.5	18.7	26.7
Georgia	2,474	10,661,208	22.5	21.6	23.4
Hawaii	426	1,566,191	22.5	20.4	24.8
North Carolina	2,780	11,004,782	22.5	21.7	23.4
Maryland	1,583	6,566,116	22.2	21.1	23.3
Florida	6,631	23,829,423	21.8	21.3	22.4
Washington	1,847	7,751,543	21.6	20.6	22.6
Montana	303	1,152,318	21.5	19.1	24.1
Tennessee	1,760	7,232,330	21.2	20.2	22.2
Rhode Island	291	1,178,881	21.1	18.7	23.8
Texas	5,798	27,183,890	21.0	20.4	21.5
Kansas	724	3,010,071	20.9	19.4	22.5
Ohio	3,161	12,827,335	20.9	20.2	21.7
North Dakota	182	751,672	20.8	17.8	24.2
West Virginia	541	2,153,056	20.8	19.1	22.7
Arkansas	785	3,203,525	20.7	19.2	22.2
Oregon	1,087	4,512,629	20.7	19.5	22.0
Virginia	2,068	9,078,070	20.7	19.8	21.7
New Hampshire	378	1,569,486	20.6	18.5	22.9

Wyoming	143	618,063	20.4	17.1	24.2
Massachusetts	1,746	7,549,758	20.3	19.3	21.3
Missouri	1,562	6,604,749	20.2	19.2	21.3
New Mexico	529	2,210,981	20.2	18.5	22.1
California	8,633	40,556,874	19.9	19.5	20.4
Nevada	675	3,130,791	19.5	18.1	21.1
Arizona	1,693	7,265,402	19.4	18.4	20.3
Oklahoma	903	4,046,170	19.3	18.0	20.6
Utah	497	2,629,635	19.2	17.5	21.0
Colorado	1,140	5,746,551	18.9	17.8	20.0
Vermont	168	730,536	18.5	15.8	21.7

Supplementary Table 5. Annual and average annual percent change in age-standardized pancreatic cancer incidence rates over time among the U.S. population over 35 years-old, by state

State	Joinpoint segment Year start	Year end	APC (95% CI)	Joinpoint segment Year start	Year end	APC (95% CI)
Alaska	2001	2016	0.38 (-1.15, 1.93)	2001	2016	0.38 (-1.15, 1.93)
Alabama	2001	2016	1.52 (0.78, 2.27)	2001	2016	1.52 (0.78, 2.27)
Arkansas	2001	2016	0.65 (0.10, 1.21)	2001	2016	0.65 (0.10, 1.21)
Arizona	2001	2004	-2.33 (-7.10, 2.70)	2001	2016	0.40 (-0.74, 1.56)
	2004	2012	2.71 (1.56, 3.86)			
	2012	2016	-2.06 (-4.49, 0.44)			
California	2001	2008	1.24 (0.43, 2.06)	2001	2016	-0.07 (-0.48, 0.34)
	2008	2016	-1.20 (-1.69, -0.71)			
Colorado	2001	2016	-0.11 (-0.68, 0.47)	2001	2016	-0.11 (-0.68, 0.47)
Connecticut	2001	2009	2.03 (0.80, 3.28)	2001	2016	1.08 (-0.51, 2.69)
	2009	2013	-3.51 (-8.55, 1.80)			
	2013	2016	4.87 (-0.28, 10.28)			
Delaware	2001	2016	1.63 (0.70, 2.58)	2001	2016	1.63 (0.70, 2.58)
Florida	2001	2016	0.77 (0.42, 1.12)	2001	2016	0.77 (0.42, 1.12)
Georgia	2001	2016	0.94 (0.48, 1.41)	2001	2016	0.94 (0.48, 1.41)
Hawaii	2001	2016	0.20 (-0.63, 1.04)	2001	2016	0.20 (-0.63, 1.04)
Idaho	2001	2016	0.76 (-0.12, 1.65)	2001	2016	0.76 (-0.12, 1.65)
Illinois	2001	2005	1.93 (-0.08, 3.98)	2001	2016	0.30 (-0.24, 0.84)
	2005	2016	-0.29 (-0.69, 0.11)			
Indiana	2001	2016	1.10 (0.74, 1.45)	2001	2016	1.10 (0.74, 1.45)
Iowa	2001	2016	0.90 (0.43, 1.37)	2001	2016	0.90 (0.43, 1.37)
Kansas	2001	2016	1.19 (0.37, 2.02)	2001	2016	1.19 (0.37, 2.02)
Kentucky	2001	2016	1.18 (0.64, 1.72)	2001	2016	1.18 (0.64, 1.72)
Louisiana	2001	2016	0.66 (0.06, 1.27)	2001	2016	0.66 (0.06, 1.27)
Massachussetts	2001	2016	-0.32 (-1.05, 0.41)	2001	2016	-0.32 (-1.05, 0.41)
Maryland	2001	2016	0.26 (-0.22, 0.74)	2001	2016	0.26 (-0.22, 0.74)
Maine	2001	2016	0.33 (-0.57, 1.25)	2001	2016	0.33 (-0.57, 1.25)
Michigan	2001	2016	0.23 (-0.23, 0.70)	2001	2016	0.23 (-0.23, 0.70)
Minnesota	2001	2016	2.66 (2.00, 3.32)	2001	2016	2.66 (2.00, 3.32)
Missouri	2001	2010	1.50 (0.02, 3.01)	2001	2016	0.13 (-1.06, 1.33)
	2010	2016	-1.89 (-4.32, 0.61)			
Mississippi	2003	2016	2.99 (2.08, 3.90)	2003	2016	2.99 (2.08, 3.90)
Montana	2001	2016	1.16 (-0.08, 2.41)	2001	2016	1.16 (-0.08, 2.41)
North Carolina	2001	2016	1.12 (0.63, 1.62)	2001	2016	1.12 (0.63, 1.62)
North Dakota	2001	2016	0.81 (-0.22, 1.86)	2001	2016	0.81 (-0.22, 1.86)
Nebraska	2001	2016	1.58 (0.76, 2.40)	2001	2016	1.58 (0.76, 2.40)
New Hampshire	2001	2005	6.62 (-1.51, 15.41)	2001	2016	0.74 (-1.37, 2.89)
	2005	2016	-1.32 (-2.75, 0.13)			
New Jersey	2001	2009	1.54 (0.54, 2.54)	2001	2016	0.56 (-0.10, 1.22)
	2009	2016	-0.55 (-1.66, 0.56)			
New Mexico	2001	2016	1.05 (0.11, 1.99)	2001	2016	1.05 (0.11, 1.99)
Nevada	2001	2016	-0.85 (-1.68, -0.01)	2001	2016	-0.85 (-1.68, -0.01)
New York	2001	2009	0.88 (0.42, 1.34)	2001	2016	0.41 (0.09, 0.72)
	2009	2016	-0.13 (-0.67, 0.41)			
Ohio	2001	2007	3.43 (1.76, 5.14)	2001	2016	1.01 (-0.68, 2.73)
	2007	2010	-3.51 (-11.77, 5.53)			
	2010	2016	0.93 (-0.44, 2.31)			
Oklahoma	2001	2016	-0.03 (-0.67, 0.61)	2001	2016	-0.03 (-0.67, 0.61)
Oregon	2001	2016	0.30 (-0.17, 0.77)	2001	2016	0.30 (-0.17, 0.77)

Pennsylvania	2001	2016	0.94 (0.63, 1.26)	2001	2016	0.94 (0.63, 1.26)
Rhode Island	2001	2016	0.53 (-0.70, 1.76)	2001	2016	0.53 (-0.70, 1.77)
South Carolina	2001	2016	1.01 (0.52, 1.50)	2001	2016	1.01 (0.52, 1.50)
South Dakota	2001	2016	1.45 (0.04, 2.88)	2001	2016	1.45 (0.04, 2.88)
Tennessee	2001	2007	4.02 (2.45, 5.61)	2001	2016	2.21 (1.54, 2.88)
	2007	2016	1.03 (0.32, 1.73)			
Texas	2001	2016	0.65 (0.32, 0.98)	2001	2016	0.65 (0.32, 0.98)
Utah	2001	2016	0.61 (-0.37, 1.61)	2001	2016	0.61 (-0.37, 1.61)
Virginia	2001	2004	17.10 (4.77, 30.89)	2001	2016	3.11 (0.94, 5.32)
	2004	2016	-0.12 (-1.19, 0.96)			
Vermont	2001	2016	0.34 (-1.60, 2.30)	2001	2016	0.34 (-1.60, 2.30)
Washington	2001	2016	-0.22 (-0.67, 0.22)	2001	2016	-0.22 (-0.67, 0.22)
Wisconsin	2001	2016	1.25 (0.63, 1.87)	2001	2016	1.25 (0.63, 1.87)
West Virginia	2001	2016	1.49 (0.71, 2.28)	2001	2016	1.49 (0.71, 2.28)
Wyoming	2001	2016	-0.16 (-2.02, 1.73)	2001	2016	-0.16 (-2.02, 1.73)

Supplementary Table 6. Annual and average annual percent change in age-standardized pancreatic cancer incidence rates over time among non-Hispanic whites over 35 years-old, by state

State	Joinpoint segment Year start	Year end	APC (95% CI)	Joinpoint segment Year start	Year end	APC (95% CI)
Alaska	2001	2016	0.19 (-1.04, 1.43)	2001	2016	0.19 (-1.04, 1.43)
Alabama	2001	2016	1.61 (0.88, 2.35)	2001	2016	1.61 (0.88, 2.35)
Arkansas	2001	2016	0.57 (-0.09, 1.23)	2001	2016	0.57 (-0.09, 1.23)
Arizona	2001	2012	1.93 (1.22, 2.64)	2001	2016	0.87 (0.03, 1.72)
	2012	2016	-1.97 (-4.85, 0.99)			
California	2001	2008	1.54 (0.79, 2.29)	2001	2016	0.05 (-0.36, 0.47)
	2008	2016	-1.23 (-1.82, -0.64)			
Colorado	2001	2016	0.07 (-0.46, 0.60)	2001	2016	0.07 (-0.46, 0.60)
Connecticut	2001	2009	2.35 (1.23, 3.48)	2001	2016	1.13 (-0.70, 2.99)
	2009	2012	-4.65 (-13.29, 4.85)			
	2013	2016	3.18 (0.12, 6.33)			
Delaware	2001	2016	1.75 (0.70, 2.81)	2001	2016	1.75 (0.70, 2.81)
Florida	2001	2016	0.94 (0.60, 1.27)	2001	2016	0.94 (0.60, 1.27)
Georgia	2001	2016	1.37 (0.81, 1.92)	2001	2016	1.37 (0.81, 1.92)
Hawaii	2001	2016	-0.03 (-1.90, 1.88)	2001	2016	-0.03 (-1.90, 1.88)
Idaho	2001	2016	0.76 (-0.22, 1.75)	2001	2016	0.76 (-0.22, 1.75)
Illinois	2001	2016	0.38 (0.04, 0.72)	2001	2016	0.38 (0.04, 0.72)
Indiana	2001	2016	1.25 (0.92, 1.58)	2001	2016	1.25 (0.92, 1.58)
Iowa	2001	2016	1.00 (0.53, 1.47)	2001	2016	1.00 (0.53, 1.47)
Kansas	2001	2016	1.27 (0.40, 2.14)	2001	2016	1.27 (0.40, 2.14)
Kentucky	2001	2016	1.31 (0.80, 1.83)	2001	2016	1.31 (0.80, 1.83)
Louisiana	2001	2016	1.23 (0.60, 1.86)	2001	2016	1.23 (0.60, 1.86)
Massachusetts	2001	2016	-0.33 (-1.11, 0.45)	2001	2016	-0.33 (-1.11, 0.45)
Maryland	2001	2016	0.44 (-0.07, 0.95)	2001	2016	0.44 (-0.07, 0.95)
Maine	2001	2016	0.33 (-0.56, 1.23)	2001	2016	0.33 (-0.56, 1.23)
Michigan	2001	2016	0.16 (-0.32, 0.64)	2001	2016	0.16 (-0.32, 0.64)
Minnesota	2001	2016	2.70 (2.10, 3.30)	2001	2016	2.70 (2.10, 3.30)
Missouri	2001	2016	0.32 (-0.36, 1.00)	2001	2016	0.32 (-0.36, 1.00)
Mississippi	2003	2016	3.20 (2.02, 4.38)	2003	2016	3.20 (2.02, 4.38)
Montana	2001	2016	1.19 (-0.21, 2.60)	2001	2016	1.19 (-0.21, 2.60)
North Carolina	2001	2016	1.19 (0.75, 1.64)	2001	2016	1.19 (0.75, 1.64)
North Dakota	2001	2016	0.73 (-0.28, 1.75)	2001	2016	0.73 (-0.28, 1.75)
Nebraska	2001	2016	1.65 (0.69, 2.62)	2001	2016	1.65 (0.69, 2.62)
New Hampshire	2001	2016	0.21 (-0.82, 1.24)	2001	2016	0.21 (-0.82, 1.24)
New Jersey	2001	2016	0.79 (0.37, 1.21)	2001	2016	0.79 (0.37, 1.21)
New Mexico	2001	2016	1.26 (0.18, 2.34)	2001	2016	1.26 (0.18, 2.34)
Nevada	2001	2016	-0.51 (-1.43, 0.43)	2001	2016	-0.85 (-1.68, -0.01)
New York	2001	2016	0.50 (0.21, 0.79)	2001	2016	0.50 (0.21, 0.79)
Ohio	2001	2007	3.35 (1.81, 4.91)	2001	2016	1.14 (-0.46, 2.76)
	2007	2010	-3.23 (-11.00, 5.21)			
	2010	2016	1.19 (-0.28, 2.67)			
Oklahoma	2001	2016	-0.06 (-0.74, 0.63)	2001	2016	-0.06 (-0.74, 0.63)
Oregon	2001	2014	0.68 (0.17, 1.19)	2001	2016	-0.17 (-1.30, 0.96)
	2014	2016	-5.55 (-13.63, 3.30)			
Pennsylvania	2001	2016	1.11 (0.83, 1.38)	2001	2016	1.11 (0.83, 1.38)
Rhode Island	2001	2016	0.93 (-0.24, 2.11)	2001	2016	0.93 (-0.24, 2.11)
South Carolina	2001	2016	1.07 (0.47, 1.67)	2001	2016	1.07 (0.47, 1.67)
South Dakota	2001	2016	1.34 (-0.09, 2.78)	2001	2016	1.34 (-0.09, 2.78)
Tennessee	2001	2007	4.41 (2.43, 6.43)	2001	2016	2.31 (1.46, 3.15)
	2007	2016	0.93 (0.05, 1.81)			

Texas	2001	2016	0.91 (0.53, 1.29)	2001	2016	0.91 (0.53, 1.29)
Utah	2001	2016	0.53 (-0.64, 1.71)	2001	2016	0.53 (-0.64, 1.71)
Virginia	2001	2004	16.79 (3.64, 31.61)	2001	2016	3.05 (0.75, 5.41)
	2004	2016	-0.12 (-1.20, 0.97)			
Vermont	2001	2016	0.24 (-1.69, 2.20)	2001	2016	0.24 (-1.69, 2.20)
Washington	2001	2016	-0.10 (-0.57, 0.36)	2001	2016	-0.10 (-0.57, 0.36)
Wisconsin	2001	2016	1.09 (0.44, 1.75)	2001	2016	1.09 (0.44, 1.75)
West Virginia	2001	2016	1.54 (0.74, 2.35)	2001	2016	1.54 (0.74, 2.35)
Wyoming	2001	2016	-0.16 (-2.05, 1.77)	2001	2016	-0.16 (-2.05, 1.77)

Supplementary Table 7. Annual and average annual percent change in age-standardized pancreatic cancer incidence rates over time among non-Hispanic blacks over 35 years-old, by state

State	Joinpoint segment Year start	Joinpoint segment Year end	APC (95% CI)	Joinpoint segment Year start	Joinpoint segment Year end	AAPC (95% CI)
Alaska	-	-	-	-	-	-
Alabama	2001	2016	1.41 (0.26, 2.57)	2001	2016	1.41 (0.26, 2.57)
Arkansas	2001	2016	0.84 (-0.56, 2.27)	2001	2016	0.84 (-0.56, 2.27)
Arizona	-	-	-	-	-	-
California	2001	2005	3.95 (-2.20, 10.48)	2001	2016	-0.39 (-2.02, 1.27)
	2005	2016	-1.92 (-3.11, -0.72)			
Colorado	-	-	-	-	-	-
Connecticut	2001	2016	-1.96 (-4.05, 0.18)	2001	2016	-1.96 (-4.05, 0.18)
Delaware	-	-	-	-	-	-
Florida	2001	2016	0.39 (-0.62, 1.42)	2001	2016	0.39 (-0.62, 1.42)
Georgia	2001	2016	0.06 (-0.49, 0.60)	2001	2016	0.06 (-0.49, 0.60)
Hawaii	-	-	-	-	-	-
Idaho	-	-	-	-	-	-
Illinois	2001	2016	-0.44 (-1.26, 0.39)	2001	2016	-0.44 (-1.26, 0.39)
Indiana	2001	2016	0.06 (-1.15, 1.30)	2001	2016	0.06 (-1.15, 1.30)
Iowa	-	-	-	-	-	-
Kansas	-	-	-	-	-	-
Kentucky	2001	2016	0.49 (-1.37, 2.38)	2001	2016	0.49 (-1.37, 2.38)
Louisiana	2001	2016	-0.67 (-1.75, 0.43)	2001	2016	-0.67 (-1.75, 0.43)
Massachussetts	2001	2016	-0.50 (-2.37, 1.40)	2001	2016	-0.67 (-1.75, 0.43)
Maryland	2001	2016	0.13 (-0.86, 1.13)	2001	2016	0.13 (-0.86, 1.13)
Maine	-	-	-	-	-	-
Michigan	2001	2006	4.29 (0.12, 8.64)	2001	2016	0.69 (-0.74, 2.13)
	2006	2016	-1.07 (-2.30, 0.17)			
Minnesota	-	-	-	-	-	-
Missouri	2001	2016	0.92 (-0.87, 2.75)	2001	2016	0.92 (-0.87, 2.75)
Mississippi	2003	2016	2.52 (1.13, 3.94)	2003	2016	2.52 (1.13, 3.94)
Montana	-	-	-	-	-	-
North Carolina	2001	2016	0.46 (-0.52, 1.45)	2001	2016	0.46 (-0.52, 1.45)
North Dakota	-	-	-	-	-	-
Nebraska	-	-	-	-	-	-
New Hampshire	-	-	-	-	-	-
New Jersey	2001	2008	4.08 (0.60, 7.67)	2001	2016	0.79 (-1.02, 2.63)
	2008	2016	-2.00 (-4.33, 0.38)			
New Mexico	-	-	-	-	-	-
Nevada	-	-	-	-	-	-
New York	2001	2016	0.44 (-0.34, 1.22)	2001	2016	0.44 (-0.34, 1.22)
Ohio	2001	2008	2.83 (-0.03, 5.77)	2001	2016	-0.12 (-1.65, 1.44)
	2008	2016	-2.63 (-4.68, -0.53)			
Oklahoma	2001	2016	-2.40 (-4.33, -0.43)	2001	2016	-2.40 (-4.33, -0.43)
Oregon	-	-	-	-	-	-
Pennsylvania	2001	2016	0.53 (-0.58, 1.65)	2001	2016	0.53 (-0.58, 1.65)
Rhode Island	-	-	-	-	-	-
South Carolina	2001	2016	0.92 (-0.39, 2.25)	2001	2016	0.92 (-0.39, 2.25)
South Dakota	-	-	-	-	-	-
Tennessee	2001	2016	1.67 (0.55, 2.81)	2001	2016	1.67 (0.55, 2.81)
Texas	2001	2016	-0.07 (-0.70, 0.56)	2001	2016	-0.07 (-0.70, 0.56)
Utah	-	-	-	-	-	-
Virginia	2001	2005	14.05 (-1.12, 31.54)	2001	2016	3.53 (-0.25, 7.46)
	2004	2016	-0.05 (-2.38, 2.34)			

Vermont	-	-	-	-	-	-
Washington	2001	2016	-1.64 (-3.79, 0.55)	2001	2016	-1.64 (-3.79, 0.55)
Wisconsin	2001	2016	2.18 (0.83, 3.56)	2001	2016	2.18 (0.83, 3.56)
West Virginia	-	-	-	-	-	-
Wyoming	-	-	-	-	-	-

Supplementary Table 8. Annual and average annual percent change in age-standardized pancreatic cancer incidence rates over time among Hispanics over 35 years-old, by state

State	Joinpoint segment Year start	Year end	APC (95% CI)	Joinpoint segment Year start	Year end	AAPC (95% CI)
Alaska	-	-	-	-	-	-
Alabama	-	-	-	-	-	-
Arkansas	-	-	-	-	-	-
Arizona	2001	2016	0.76 (-0.93, 2.49)	2001	2016	0.76 (-0.93, 2.49)
California	2001	2016	-0.29 (-0.80, 0.22)	2001	2016	-0.29 (-0.80, 0.22)
Colorado	2001	2016	-1.72 (-4.21, 0.84)	2001	2016	-1.72 (-4.21, 0.84)
Connecticut	-	-	-	-	-	-
Delaware	-	-	-	-	-	-
Florida	2001	2016	0.33 (-0.38, 1.04)	2001	2016	0.33 (-0.38, 1.04)
Georgia	-	-	-	-	-	-
Hawaii	-	-	-	-	-	-
Idaho	-	-	-	-	-	-
Illinois	2001	2006	8.56 (-1.95, 20.20)	2001	2016	1.52 (-1.88, 5.03)
	2005	2016	-1.83 (-4.38, 0.79)			
Indiana	-	-	-	-	-	-
Iowa	-	-	-	-	-	-
Kansas	-	-	-	-	-	-
Kentucky	-	-	-	-	-	-
Louisiana	-	-	-	-	-	-
Massachussetts	-	-	-	-	-	-
Maryland	-	-	-	-	-	-
Maine	-	-	-	-	-	-
Michigan	-	-	-	-	-	-
Minnesota	-	-	-	-	-	-
Missouri	-	-	-	-	-	-
Mississippi	-	-	-	-	-	-
Montana	-	-	-	-	-	-
North Carolina	-	-	-	-	-	-
North Dakota	-	-	-	-	-	-
Nebraska	-	-	-	-	-	-
New Hampshire	-	-	-	-	-	-
New Jersey	2001	2016	-0.10 (-1.41, 1.22)	2001	2016	-0.10 (-1.41, 1.22)
New Mexico	2001	2016	0.51 (-1.10, 2.14)	2001	2016	0.51 (-1.10, 2.14)
Nevada	-	-	-	-	-	-
New York	2001	2016	0.63 (-0.12, 1.39)	2001	2016	0.41 (0.09, 0.72)
Ohio	-	-	-	-	-	-
Oklahoma	-	-	-	-	-	-
Oregon	-	-	-	-	-	-
Pennsylvania	-	-	-	-	-	-
Rhode Island	-	-	-	-	-	-
South Carolina	-	-	-	-	-	-
South Dakota	-	-	-	-	-	-
Tennessee	-	-	-	-	-	-
Texas	2001	2016	0.29 (-0.28, 0.87)	2001	2016	0.29 (-0.28, 0.87)
Utah	-	-	-	-	-	-
Virginia	-	-	-	-	-	-
Vermont	-	-	-	-	-	-
Washington	-	-	-	-	-	-
Wisconsin	-	-	-	-	-	-
West Virginia	-	-	-	-	-	-

Wyoming	-	-	-	-	-	-
